# 3,5-Bis(adamantan-1-yl)-1-meth­oxy­benzene

**DOI:** 10.1107/S1600536812011622

**Published:** 2012-03-24

**Authors:** Peng-Fei Li, Jian-Zi Sun, Ling Wang, Ya-Li Lv, Li-Hong Liu

**Affiliations:** a Pharmacy Department of The Second Artillery General Hospital, Beijing 100088, People’s Republic of China

## Abstract

In title compound, C_27_H_36_O, all cyclo­hexane rings within the adamantyl groups adopt chair conformations. There are no obvious inter­molecular hydrogen bonds in the structure, so that van der Waals attractions stabilize the crystal.

## Related literature

For applications of liquid materials, see: Binnemans (2005 ▶); Vyklický *et al.* (2003 ▶). For bond-length data, see: Allen *et al.* (1987 ▶); Pröhl *et al.* (1999 ▶).
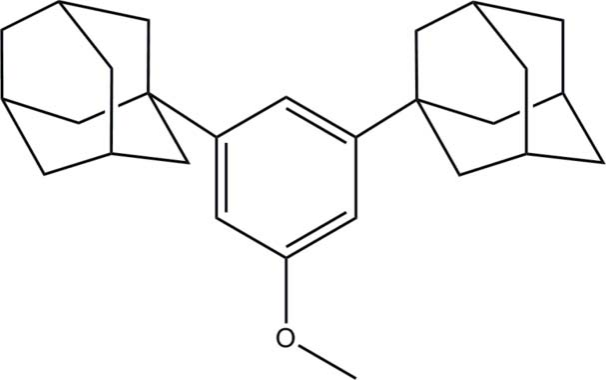


## Experimental

### 

#### Crystal data

C_27_H_36_O
*M* *_r_* = 376.56
Monoclinic, 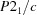

*a* = 10.4672 (12) Å
*b* = 20.170 (2) Å
*c* = 10.9202 (13) Å
β = 117.909 (3)°
*V* = 2037.4 (4) Å^3^
*Z* = 4
Mo *K*α radiation
μ = 0.07 mm^−1^
*T* = 113 K
0.20 × 0.18 × 0.10 mm

#### Data collection

Rigaku Saturn CCD area-detector diffractometer
Absorption correction: multi-scan (*CrystalClear-SM Expert*; Rigaku/MSC, 2009) ▶ *T*_min_ = 0.986, *T*_max_ = 0.993
20680 measured reflections
4867 independent reflections
3768 reflections with *I* > 2σ(*I*)
*R*_int_ = 0.047

#### Refinement

*R*[*F* ^2^ > 2σ(*F* ^2^)] = 0.059
*wR*(*F* ^2^) = 0.143
*S* = 1.09
4867 reflections
254 parameters
H-atom parameters constrained
Δρ_max_ = 0.26 e Å^−3^
Δρ_min_ = −0.22 e Å^−3^

### 

Data collection: *CrystalClear-SM Expert* (Rigaku/MSC, 2009) ▶; cell refinement: *CrystalClear-SM Expert*; data reduction: *CrystalClear-SM Expert*; program(s) used to solve structure: *SHELXS97* (Sheldrick, 2008 ▶); program(s) used to refine structure: *SHELXL97* (Sheldrick, 2008 ▶); molecular graphics: *SHELXTL* (Sheldrick, 2008 ▶); software used to prepare material for publication: *SHELXTL*.

## Supplementary Material

Crystal structure: contains datablock(s) I, global. DOI: 10.1107/S1600536812011622/hg5189sup1.cif

Structure factors: contains datablock(s) I. DOI: 10.1107/S1600536812011622/hg5189Isup2.hkl

Supplementary material file. DOI: 10.1107/S1600536812011622/hg5189Isup3.cml

Additional supplementary materials:  crystallographic information; 3D view; checkCIF report

## Figures and Tables

**Fig. 1.**
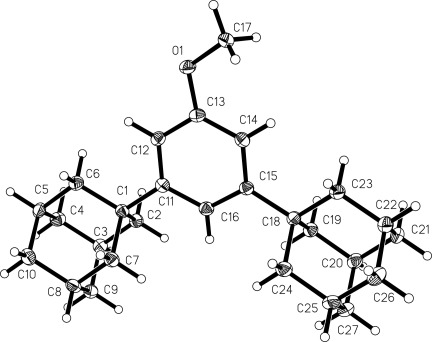
View of the title compound, with displacement ellipsoids drawn at the 40% probability level.

## supplementary crystallographic information

### Comment

Liquid crystals are an important class of functional materials (Binnemans, 2005). During the preparation of highly orderly adamantane-bearing liquid materials (Vyklický *et al.*, 2003), the title compound was prepared as a key intermediate.

In title compound, C_27_H_36_O, all bond lengths and angles in the molecular are normal (Allen *et al.*, 1987) and in a good agreement with those reported previously (Pröhl *et al.*, 1999). All cyclohexane rings within adamantylamine adopt chair conformation. There are no obvious intermolecular hydrogen bonds founded in structure with Van der Waasl attractions stabilizing the crystal.

### Experimental

A dried 100-ml round-bottomed flask was charged with 4.30 g (20 mmol) of 1-adamantyl bromide, 1.08 g (10 mmol) of anisole and 15 ml of dried dichloromethane. The mixture was stirred on an ice-water bath, followed by addition of 1.33 g (10 mmol) of anhydrous aluminium chloride in a portionwise manner. After addition, the reaction mixture was stirred at room temperature for 1 h and at reflux overnight, and poured into 300 ml of ice-water. The mixture thus formed was exacted with three 50-ml portions of dichloromethane, and the combined exacts were washed with saturated brine, dried over sodium sulfate and evaporated on a rotary evaporator to afford the crude title compound. Pure title compound was obtained by column chromatography. Crystals suitable for X-ray diffraction were obtained through slow evaporation of a solution of the pure title compound in dichloromethane/petroleum ether (1/10 by volume).

### Refinement

All H atoms were found on difference maps, with C—H = 0.95–1.00, and included in the final cycles of refinement using a riding model, with *U*_iso_(H) = 1.2*U*_eq_(C) and 1.5*U*_eq_(C) for methyl H atoms.

### Crystal data

**Table:**

C_27_H_36_O	*F*(000) = 824
*M**_r_* = 376.56	*D*_x_ = 1.228 Mg m^−^^3^
Monoclinic, *P*2_1_/*c*	Mo *K*α radiation, λ = 0.71073 Å
Hall symbol: -P 2ybc	Cell parameters from 6285 reflections
*a* = 10.4672 (12) Å	θ = 2.0–27.9°
*b* = 20.170 (2) Å	µ = 0.07 mm^−^^1^
*c* = 10.9202 (13) Å	*T* = 113 K
β = 117.909 (3)°	Prism, colorless
*V* = 2037.4 (4) Å^3^	0.20 × 0.18 × 0.10 mm
*Z* = 4	

### Data collection

**Table:**

Rigaku Saturn CCD area-detector diffractometer	4867 independent reflections
Radiation source: rotating anode	3768 reflections with *I* > 2σ(*I*)
Multilayer monochromator	*R*_int_ = 0.047
Detector resolution: 14.63 pixels mm^-1^	θ_max_ = 27.9°, θ_min_ = 2.0°
ω and φ scans	*h* = −13→12
Absorption correction: multi-scan (*CrystalClear-SM Expert*; Rigaku/MSC, 2009)	*k* = −26→26
*T*_min_ = 0.986, *T*_max_ = 0.993	*l* = −14→14
20680 measured reflections	

### Refinement

**Table:**

Refinement on *F*^2^	Primary atom site location: structure-invariant direct methods
Least-squares matrix: full	Secondary atom site location: difference Fourier map
*R*[*F*^2^ > 2σ(*F*^2^)] = 0.059	Hydrogen site location: inferred from neighbouring sites
*wR*(*F*^2^) = 0.143	H-atom parameters constrained
*S* = 1.09	*w* = 1/[σ^2^(*F*_o_^2^) + (0.0639*P*)^2^ + 0.0855*P*] where *P* = (*F*_o_^2^ + 2*F*_c_^2^)/3
4867 reflections	(Δ/σ)_max_ = 0.001
254 parameters	Δρ_max_ = 0.26 e Å^−^^3^
0 restraints	Δρ_min_ = −0.22 e Å^−^^3^

### Special details

**Table:**

Geometry. All e.s.d.'s (except the e.s.d. in the dihedral angle between two l.s. planes) are estimated using the full covariance matrix. The cell e.s.d.'s are taken into account individually in the estimation of e.s.d.'s in distances, angles and torsion angles; correlations between e.s.d.'s in cell parameters are only used when they are defined by crystal symmetry. An approximate (isotropic) treatment of cell e.s.d.'s is used for estimating e.s.d.'s involving l.s. planes.
Refinement. Refinement of *F*^2^ against ALL reflections. The weighted *R*-factor *wR* and goodness of fit *S* are based on *F*^2^, conventional *R*-factors *R* are based on *F*, with *F* set to zero for negative *F*^2^. The threshold expression of *F*^2^ > σ(*F*^2^) is used only for calculating *R*-factors(gt) *etc*. and is not relevant to the choice of reflections for refinement. *R*-factors based on *F*^2^ are statistically about twice as large as those based on *F*, and *R*- factors based on ALL data will be even larger.

### Fractional atomic coordinates and isotropic or equivalent isotropic displacement parameters (Å^2^)

**Table:**

	*x*	*y*	*z*	*U*_iso_*/*U*_eq_	
O1	1.10593 (10)	0.27919 (5)	0.58496 (10)	0.0250 (3)	
C1	1.11441 (15)	0.47721 (7)	0.33134 (14)	0.0197 (3)	
C2	1.12790 (16)	0.47313 (8)	0.19713 (15)	0.0222 (3)	
H2A	1.1873	0.4341	0.2016	0.027*	
H2B	1.0307	0.4671	0.1172	0.027*	
C3	1.19747 (16)	0.53597 (8)	0.17552 (15)	0.0232 (3)	
H3	1.2041	0.5322	0.0874	0.028*	
C4	1.34892 (16)	0.54442 (8)	0.29754 (16)	0.0266 (4)	
H4A	1.4097	0.5058	0.3028	0.032*	
H4B	1.3947	0.5848	0.2841	0.032*	
C5	1.33776 (16)	0.55017 (8)	0.43180 (15)	0.0242 (3)	
H5	1.4366	0.5556	0.5119	0.029*	
C6	1.26680 (16)	0.48728 (8)	0.45259 (15)	0.0235 (3)	
H6A	1.3274	0.4483	0.4600	0.028*	
H6B	1.2608	0.4910	0.5401	0.028*	
C7	1.02432 (16)	0.53955 (8)	0.32272 (16)	0.0236 (3)	
H7A	0.9256	0.5346	0.2446	0.028*	
H7B	1.0162	0.5436	0.4091	0.028*	
C8	1.09363 (17)	0.60227 (8)	0.30197 (16)	0.0247 (4)	
H8	1.0333	0.6417	0.2965	0.030*	
C9	1.10540 (16)	0.59624 (8)	0.16823 (16)	0.0256 (4)	
H9A	1.1499	0.6369	0.1543	0.031*	
H9B	1.0076	0.5915	0.0885	0.031*	
C10	1.24469 (17)	0.61016 (8)	0.42377 (16)	0.0279 (4)	
H10A	1.2381	0.6141	0.5110	0.033*	
H10B	1.2899	0.6511	0.4119	0.033*	
C11	1.03923 (15)	0.41538 (7)	0.34680 (14)	0.0195 (3)	
C12	1.10490 (15)	0.37136 (8)	0.45648 (15)	0.0206 (3)	
H12	1.2027	0.3782	0.5237	0.025*	
C13	1.02926 (15)	0.31740 (8)	0.46913 (14)	0.0208 (3)	
C14	0.88604 (15)	0.30602 (8)	0.37180 (15)	0.0213 (3)	
H14	0.8356	0.2688	0.3811	0.026*	
C15	0.81658 (15)	0.34982 (7)	0.25986 (15)	0.0197 (3)	
C16	0.89534 (15)	0.40299 (8)	0.24905 (15)	0.0214 (3)	
H16	0.8497	0.4321	0.1722	0.026*	
C17	1.03023 (17)	0.22817 (8)	0.61397 (16)	0.0259 (4)	
H17A	0.9476	0.2473	0.6205	0.039*	
H17B	1.0952	0.2068	0.7020	0.039*	
H17C	0.9957	0.1952	0.5394	0.039*	
C18	0.65703 (15)	0.34265 (7)	0.15266 (15)	0.0199 (3)	
C19	0.64195 (15)	0.33224 (8)	0.00651 (15)	0.0226 (3)	
H19A	0.6889	0.3695	−0.0160	0.027*	
H19B	0.6920	0.2908	0.0052	0.027*	
C20	0.48285 (16)	0.32808 (8)	−0.10310 (16)	0.0253 (4)	
H20	0.4764	0.3213	−0.1966	0.030*	
C21	0.41172 (17)	0.26967 (8)	−0.06915 (16)	0.0282 (4)	
H21A	0.3089	0.2664	−0.1398	0.034*	
H21B	0.4607	0.2279	−0.0705	0.034*	
C22	0.42223 (16)	0.27972 (8)	0.07408 (17)	0.0277 (4)	
H22	0.3760	0.2414	0.0962	0.033*	
C23	0.58103 (15)	0.28475 (8)	0.18362 (16)	0.0239 (3)	
H23A	0.6312	0.2428	0.1858	0.029*	
H23B	0.5869	0.2912	0.2760	0.029*	
C24	0.57376 (16)	0.40698 (8)	0.14937 (16)	0.0247 (4)	
H24A	0.5802	0.4143	0.2417	0.030*	
H24B	0.6193	0.4453	0.1283	0.030*	
C25	0.41461 (16)	0.40257 (8)	0.04035 (16)	0.0276 (4)	
H25	0.3638	0.4445	0.0406	0.033*	
C26	0.34348 (17)	0.34392 (9)	0.07346 (18)	0.0318 (4)	
H26A	0.2406	0.3409	0.0031	0.038*	
H26B	0.3479	0.3505	0.1652	0.038*	
C27	0.40500 (17)	0.39234 (8)	−0.10303 (16)	0.0276 (4)	
H27A	0.3024	0.3898	−0.1744	0.033*	
H27B	0.4502	0.4303	−0.1255	0.033*	

### Atomic displacement parameters (Å^2^)

**Table:**

	*U*^11^	*U*^22^	*U*^33^	*U*^12^	*U*^13^	*U*^23^
O1	0.0223 (6)	0.0256 (6)	0.0237 (6)	0.0005 (5)	0.0078 (5)	0.0082 (4)
C1	0.0217 (7)	0.0189 (8)	0.0187 (7)	−0.0005 (6)	0.0098 (6)	0.0002 (6)
C2	0.0236 (8)	0.0226 (8)	0.0199 (7)	0.0009 (6)	0.0099 (6)	−0.0015 (6)
C3	0.0275 (8)	0.0253 (9)	0.0191 (8)	−0.0003 (7)	0.0127 (7)	0.0011 (6)
C4	0.0249 (8)	0.0278 (9)	0.0282 (8)	0.0006 (7)	0.0133 (7)	0.0058 (7)
C5	0.0203 (8)	0.0267 (9)	0.0203 (8)	−0.0045 (7)	0.0051 (6)	0.0008 (6)
C6	0.0250 (8)	0.0247 (9)	0.0192 (7)	−0.0005 (7)	0.0090 (6)	0.0026 (6)
C7	0.0247 (8)	0.0220 (8)	0.0257 (8)	0.0010 (6)	0.0131 (7)	−0.0011 (6)
C8	0.0284 (8)	0.0180 (8)	0.0270 (8)	0.0030 (6)	0.0123 (7)	0.0000 (6)
C9	0.0268 (8)	0.0223 (9)	0.0240 (8)	−0.0008 (7)	0.0087 (7)	0.0034 (6)
C10	0.0354 (9)	0.0230 (9)	0.0248 (8)	−0.0042 (7)	0.0137 (7)	−0.0012 (6)
C11	0.0214 (8)	0.0202 (8)	0.0193 (7)	0.0005 (6)	0.0116 (6)	−0.0013 (6)
C12	0.0193 (7)	0.0230 (8)	0.0185 (7)	0.0019 (6)	0.0081 (6)	−0.0009 (6)
C13	0.0226 (8)	0.0218 (8)	0.0188 (7)	0.0040 (6)	0.0103 (6)	0.0026 (6)
C14	0.0231 (8)	0.0199 (8)	0.0229 (8)	−0.0005 (6)	0.0124 (7)	0.0001 (6)
C15	0.0205 (7)	0.0194 (8)	0.0191 (7)	0.0016 (6)	0.0091 (6)	−0.0006 (6)
C16	0.0234 (8)	0.0211 (8)	0.0192 (7)	0.0009 (6)	0.0095 (6)	0.0019 (6)
C17	0.0281 (8)	0.0209 (8)	0.0238 (8)	−0.0021 (7)	0.0079 (7)	0.0035 (6)
C18	0.0180 (7)	0.0208 (8)	0.0197 (7)	0.0002 (6)	0.0078 (6)	0.0009 (6)
C19	0.0228 (8)	0.0232 (8)	0.0213 (8)	0.0009 (6)	0.0099 (6)	−0.0002 (6)
C20	0.0245 (8)	0.0287 (9)	0.0188 (8)	0.0014 (7)	0.0069 (7)	−0.0016 (6)
C21	0.0203 (8)	0.0263 (9)	0.0280 (9)	−0.0024 (7)	0.0030 (7)	−0.0022 (7)
C22	0.0215 (8)	0.0273 (9)	0.0316 (9)	−0.0017 (7)	0.0101 (7)	0.0048 (7)
C23	0.0213 (8)	0.0239 (8)	0.0246 (8)	0.0006 (6)	0.0092 (7)	0.0045 (6)
C24	0.0261 (8)	0.0224 (8)	0.0235 (8)	0.0031 (7)	0.0098 (7)	−0.0012 (6)
C25	0.0248 (8)	0.0260 (9)	0.0311 (9)	0.0089 (7)	0.0122 (7)	0.0026 (7)
C26	0.0216 (8)	0.0405 (11)	0.0327 (9)	0.0034 (7)	0.0123 (7)	0.0018 (8)
C27	0.0233 (8)	0.0285 (9)	0.0240 (8)	0.0040 (7)	0.0053 (7)	0.0041 (6)

### Geometric parameters (Å, º)

**Table:**

O1—C13	1.3740 (17)	C14—C15	1.404 (2)
O1—C17	1.4221 (17)	C14—H14	0.9500
C1—C11	1.526 (2)	C15—C16	1.391 (2)
C1—C6	1.5367 (19)	C15—C18	1.5331 (19)
C1—C2	1.539 (2)	C16—H16	0.9500
C1—C7	1.548 (2)	C17—H17A	0.9800
C2—C3	1.534 (2)	C17—H17B	0.9800
C2—H2A	0.9900	C17—H17C	0.9800
C2—H2B	0.9900	C18—C23	1.537 (2)
C3—C4	1.530 (2)	C18—C19	1.543 (2)
C3—C9	1.530 (2)	C18—C24	1.554 (2)
C3—H3	1.0000	C19—C20	1.532 (2)
C4—C5	1.529 (2)	C19—H19A	0.9900
C4—H4A	0.9900	C19—H19B	0.9900
C4—H4B	0.9900	C20—C21	1.529 (2)
C5—C10	1.530 (2)	C20—C27	1.531 (2)
C5—C6	1.540 (2)	C20—H20	1.0000
C5—H5	1.0000	C21—C22	1.529 (2)
C6—H6A	0.9900	C21—H21A	0.9900
C6—H6B	0.9900	C21—H21B	0.9900
C7—C8	1.527 (2)	C22—C23	1.531 (2)
C7—H7A	0.9900	C22—C26	1.533 (2)
C7—H7B	0.9900	C22—H22	1.0000
C8—C10	1.525 (2)	C23—H23A	0.9900
C8—C9	1.527 (2)	C23—H23B	0.9900
C8—H8	1.0000	C24—C25	1.531 (2)
C9—H9A	0.9900	C24—H24A	0.9900
C9—H9B	0.9900	C24—H24B	0.9900
C10—H10A	0.9900	C25—C26	1.529 (2)
C10—H10B	0.9900	C25—C27	1.535 (2)
C11—C12	1.387 (2)	C25—H25	1.0000
C11—C16	1.4019 (19)	C26—H26A	0.9900
C12—C13	1.390 (2)	C26—H26B	0.9900
C12—H12	0.9500	C27—H27A	0.9900
C13—C14	1.3921 (19)	C27—H27B	0.9900
			
C13—O1—C17	117.70 (11)	C16—C15—C14	118.23 (13)
C11—C1—C6	113.19 (12)	C16—C15—C18	119.01 (13)
C11—C1—C2	110.10 (12)	C14—C15—C18	122.72 (13)
C6—C1—C2	107.86 (11)	C15—C16—C11	122.75 (14)
C11—C1—C7	109.86 (11)	C15—C16—H16	118.6
C6—C1—C7	107.59 (12)	C11—C16—H16	118.6
C2—C1—C7	108.08 (12)	O1—C17—H17A	109.5
C3—C2—C1	111.34 (12)	O1—C17—H17B	109.5
C3—C2—H2A	109.4	H17A—C17—H17B	109.5
C1—C2—H2A	109.4	O1—C17—H17C	109.5
C3—C2—H2B	109.4	H17A—C17—H17C	109.5
C1—C2—H2B	109.4	H17B—C17—H17C	109.5
H2A—C2—H2B	108.0	C15—C18—C23	113.24 (12)
C4—C3—C9	109.12 (12)	C15—C18—C19	110.50 (11)
C4—C3—C2	109.33 (12)	C23—C18—C19	108.14 (12)
C9—C3—C2	109.37 (12)	C15—C18—C24	109.73 (12)
C4—C3—H3	109.7	C23—C18—C24	107.28 (12)
C9—C3—H3	109.7	C19—C18—C24	107.76 (12)
C2—C3—H3	109.7	C20—C19—C18	111.29 (12)
C5—C4—C3	109.30 (12)	C20—C19—H19A	109.4
C5—C4—H4A	109.8	C18—C19—H19A	109.4
C3—C4—H4A	109.8	C20—C19—H19B	109.4
C5—C4—H4B	109.8	C18—C19—H19B	109.4
C3—C4—H4B	109.8	H19A—C19—H19B	108.0
H4A—C4—H4B	108.3	C21—C20—C27	109.41 (13)
C4—C5—C10	109.72 (13)	C21—C20—C19	109.10 (12)
C4—C5—C6	109.73 (13)	C27—C20—C19	109.72 (13)
C10—C5—C6	108.78 (12)	C21—C20—H20	109.5
C4—C5—H5	109.5	C27—C20—H20	109.5
C10—C5—H5	109.5	C19—C20—H20	109.5
C6—C5—H5	109.5	C22—C21—C20	109.62 (13)
C1—C6—C5	111.08 (12)	C22—C21—H21A	109.7
C1—C6—H6A	109.4	C20—C21—H21A	109.7
C5—C6—H6A	109.4	C22—C21—H21B	109.7
C1—C6—H6B	109.4	C20—C21—H21B	109.7
C5—C6—H6B	109.4	H21A—C21—H21B	108.2
H6A—C6—H6B	108.0	C21—C22—C23	109.88 (12)
C8—C7—C1	111.33 (12)	C21—C22—C26	109.04 (13)
C8—C7—H7A	109.4	C23—C22—C26	109.41 (13)
C1—C7—H7A	109.4	C21—C22—H22	109.5
C8—C7—H7B	109.4	C23—C22—H22	109.5
C1—C7—H7B	109.4	C26—C22—H22	109.5
H7A—C7—H7B	108.0	C22—C23—C18	111.28 (12)
C10—C8—C9	109.12 (13)	C22—C23—H23A	109.4
C10—C8—C7	109.13 (13)	C18—C23—H23A	109.4
C9—C8—C7	109.43 (13)	C22—C23—H23B	109.4
C10—C8—H8	109.7	C18—C23—H23B	109.4
C9—C8—H8	109.7	H23A—C23—H23B	108.0
C7—C8—H8	109.7	C25—C24—C18	111.33 (12)
C8—C9—C3	110.14 (12)	C25—C24—H24A	109.4
C8—C9—H9A	109.6	C18—C24—H24A	109.4
C3—C9—H9A	109.6	C25—C24—H24B	109.4
C8—C9—H9B	109.6	C18—C24—H24B	109.4
C3—C9—H9B	109.6	H24A—C24—H24B	108.0
H9A—C9—H9B	108.1	C26—C25—C24	109.52 (13)
C8—C10—C5	109.87 (13)	C26—C25—C27	109.30 (14)
C8—C10—H10A	109.7	C24—C25—C27	109.20 (12)
C5—C10—H10A	109.7	C26—C25—H25	109.6
C8—C10—H10B	109.7	C24—C25—H25	109.6
C5—C10—H10B	109.7	C27—C25—H25	109.6
H10A—C10—H10B	108.2	C25—C26—C22	109.53 (13)
C12—C11—C16	117.70 (14)	C25—C26—H26A	109.8
C12—C11—C1	123.09 (13)	C22—C26—H26A	109.8
C16—C11—C1	119.17 (13)	C25—C26—H26B	109.8
C11—C12—C13	120.78 (13)	C22—C26—H26B	109.8
C11—C12—H12	119.6	H26A—C26—H26B	108.2
C13—C12—H12	119.6	C20—C27—C25	109.48 (12)
O1—C13—C12	114.60 (12)	C20—C27—H27A	109.8
O1—C13—C14	124.51 (14)	C25—C27—H27A	109.8
C12—C13—C14	120.87 (13)	C20—C27—H27B	109.8
C13—C14—C15	119.65 (14)	C25—C27—H27B	109.8
C13—C14—H14	120.2	H27A—C27—H27B	108.2
C15—C14—H14	120.2		
			
C11—C1—C2—C3	177.63 (11)	C12—C13—C14—C15	−0.5 (2)
C6—C1—C2—C3	−58.42 (16)	C13—C14—C15—C16	1.1 (2)
C7—C1—C2—C3	57.63 (15)	C13—C14—C15—C18	−176.81 (13)
C1—C2—C3—C4	60.23 (16)	C14—C15—C16—C11	−1.6 (2)
C1—C2—C3—C9	−59.19 (15)	C18—C15—C16—C11	176.35 (13)
C9—C3—C4—C5	59.87 (16)	C12—C11—C16—C15	1.5 (2)
C2—C3—C4—C5	−59.71 (16)	C1—C11—C16—C15	−176.36 (13)
C3—C4—C5—C10	−59.88 (16)	C16—C15—C18—C23	−175.76 (13)
C3—C4—C5—C6	59.58 (16)	C14—C15—C18—C23	2.1 (2)
C11—C1—C6—C5	179.93 (12)	C16—C15—C18—C19	62.76 (17)
C2—C1—C6—C5	57.88 (16)	C14—C15—C18—C19	−119.35 (15)
C7—C1—C6—C5	−58.50 (15)	C16—C15—C18—C24	−55.92 (17)
C4—C5—C6—C1	−59.68 (16)	C14—C15—C18—C24	121.97 (15)
C10—C5—C6—C1	60.35 (15)	C15—C18—C19—C20	−177.44 (12)
C11—C1—C7—C8	−177.97 (12)	C23—C18—C19—C20	58.10 (16)
C6—C1—C7—C8	58.41 (15)	C24—C18—C19—C20	−57.57 (16)
C2—C1—C7—C8	−57.82 (15)	C18—C19—C20—C21	−60.08 (16)
C1—C7—C8—C10	−59.94 (16)	C18—C19—C20—C27	59.78 (17)
C1—C7—C8—C9	59.40 (16)	C27—C20—C21—C22	−60.25 (15)
C10—C8—C9—C3	59.82 (16)	C19—C20—C21—C22	59.79 (16)
C7—C8—C9—C3	−59.53 (16)	C20—C21—C22—C23	−59.49 (16)
C4—C3—C9—C8	−60.26 (16)	C20—C21—C22—C26	60.42 (15)
C2—C3—C9—C8	59.30 (15)	C21—C22—C23—C18	59.04 (17)
C9—C8—C10—C5	−59.29 (16)	C26—C22—C23—C18	−60.65 (17)
C7—C8—C10—C5	60.24 (16)	C15—C18—C23—C22	179.89 (12)
C4—C5—C10—C8	59.78 (16)	C19—C18—C23—C22	−57.31 (16)
C6—C5—C10—C8	−60.26 (15)	C24—C18—C23—C22	58.67 (16)
C6—C1—C11—C12	−2.7 (2)	C15—C18—C24—C25	178.33 (12)
C2—C1—C11—C12	118.14 (15)	C23—C18—C24—C25	−58.26 (16)
C7—C1—C11—C12	−122.94 (15)	C19—C18—C24—C25	57.97 (16)
C6—C1—C11—C16	175.04 (12)	C18—C24—C25—C26	59.68 (16)
C2—C1—C11—C16	−64.16 (16)	C18—C24—C25—C27	−59.98 (17)
C7—C1—C11—C16	54.76 (17)	C24—C25—C26—C22	−59.33 (17)
C16—C11—C12—C13	−0.8 (2)	C27—C25—C26—C22	60.27 (16)
C1—C11—C12—C13	176.96 (13)	C21—C22—C26—C25	−60.51 (16)
C17—O1—C13—C12	172.30 (13)	C23—C22—C26—C25	59.69 (17)
C17—O1—C13—C14	−5.9 (2)	C21—C20—C27—C25	59.77 (16)
C11—C12—C13—O1	−177.95 (13)	C19—C20—C27—C25	−59.90 (16)
C11—C12—C13—C14	0.3 (2)	C26—C25—C27—C20	−59.85 (16)
O1—C13—C14—C15	177.62 (14)	C24—C25—C27—C20	59.95 (17)
